# Predictive value of soluble fms‐like tyrosine kinase‐1 against placental growth factor for preeclampsia in a Chinese pregnant women population

**DOI:** 10.1002/jcla.22861

**Published:** 2019-02-13

**Authors:** Fan Yu, Qianjin Bai, Shihong Zhang, Yongmei Jiang

**Affiliations:** ^1^ Department of Laboratory Medicine West China Second University Hospital, Sichuan University Chengdu China; ^2^ Key Laboratory of Birth Defects and Related Diseases of Women and Children (Sichuan University) Ministry of Education Chengdu China; ^3^ Department of Gynaecology and Obstertrics West China Second University Hospital, Sichuan University Chengdu China

**Keywords:** placental growth factor, preeclampsia, soluble fms‐like tyrosine kinase‐1

## Abstract

**Objective:**

The purpose of the present study was to explore the predictive effects of soluble fms‐like tyrosine kinase‐1 (sFlt‐1) and placental growth factor (PlGF) for preeclampsia.

**Methods:**

A total of 1580 singleton pregnant women aged 18‐45 years were included in this study. Serum samples were collected and stored frozen during their regular obstetric examinations. A total of 48 women who were eventually diagnosed with preeclampsia among them were defined as the preeclampsia group, other 134 women who were matched with age and sample collecting gestational weeks and finally diagnosed without preeclampsia were selected as control. The concentration of sFlt‐1 and PlGF in prestored serum samples was examined. The optimal cut‐off of sFlt‐1, PlGF, and sFlt‐1/PlGF ratio in predicting preeclampsia was determined by establishing the receiver operating characteristic curve (ROC).

**Results:**

Serum PlGF levels in patients with preeclampsia were significantly lower than those in normal pregnancy (*P* < 0.05), On the contrary, sflt‐1 levels and sflt‐1/PlGF ratios were significantly higher than those in the normal pregnant women (*P* < 0.05). The ROC curve study showed that using the sFlt‐1/PlGF ratio to predict preeclampsia was better than using PlGF alone but no difference with sFlt‐1. When the cut‐off of the sFlt‐1/PlGF ratio was 26.6, the area under the ROC curve was 0.918, and high sensitivity (85.42%) and specificity (96.27%) for predicting preeclampsia were obtained.

**Conclusion:**

The cut‐off of sflt‐1/PlGF ratio determined by ROC curve has a good predictive value for the occurrence of preeclampsia.

## 1 BACKGROUND

Preeclampsia (PE) is a special disease associated with pregnancy only, mainly characterized by appearing high blood pressure and proteinuria after 20 weeks of gestation, accompanied by multiple organ damage, such as heart, brain, kidney.^1^, ^2^ Preeclampsia can cause not only thromboembolism, bleeding, convulsions, liver failure, pulmonary edema, kidney damage, renal failure, long‐term cardiovascular disease, and even death in pregnant women, but also can cause premature birth, slow growth, and low birthweight for fetus. Approximately, 60 000 pregnant women die from preeclampsia in the world every year, with an incidence of about 2% to 10% of all pregnancies, while the incidence in China is 9.4%.^3^, ^4^, ^5^ The clinical manifestations of preeclampsia are diverse, and effective diagnostic testing methods for preeclampsia are absent, which result in no reliable early warning indicators and make preeclampsia becoming a serious threat to the health of pregnant and infant. The main purpose of this study is discussing the early predictive role of sFlt‐1/PlGF ratio on preeclampsia in Western China and establishing a predictive cut‐off for the population in this area by using the ROC curve.

## 2 METHODS

### 2.1 Participants

The complete data and peripheral blood samples of 1580 singleton pregnant women aged 18‐45 years who had routine prenatal examination and did not occur preeclampsia yet between 12 and 36 gestational weeks at the Department of Gynecology and Obstetrics, West China Second University Hospital from February 2016 to October 2016 were prospectively collected as research cohort. West China Second University Hospital is a tertiary obstetrics and gynecology hospital with more than 11 000 deliveries per year. A total of 48 pregnant women among them with final diagnosis of preeclampsia were selected as study group, meanwhile, 134 healthy pregnant women among them without preeclampsia finally were matched according to age (±2 years) and sample collection gestational weeks (±1 weeks) as control group. Any pregnant women would be excluded if they exhibited any of the following conditions: pre‐existing diabetes, thyroid disorder or other endocrine disease, hypertension, preeclampsia, renal insufficiency, corticosteroid therapy, miscarried, delivered prematurely or known fetal anomaly. The diagnosis of preeclampsia was based on the development of blood pressure higher than 140/90 mm Hg on two separate occasions 6 hours apart in association with proteinuria ≥(+) by dipstick testing or proteinuria ≥300 mg per 24 hours. Superimposed preeclampsia was confirmed as a development of features of preeclampsia in the context of pre‐existing hypertension or pre‐existing proteinuria or both.^2^The research protocol was approved by the Institutional Committee for the Protection of Human Subjects (the Institutional Review Board of West China Second University Hospital, Sichuan University). Written informed consent was obtained.

### 2.2 Samples collection and examination

All peripheral blood samples were collected during regular obstetric examinations of pregnant women before diagnosis of preeclampsia. Serum was isolated by centrifuge (2700 *g* for 5 minutes) from the peripheral blood samples and stored at a temperature of −80°C. After determining the experimental group, the sflt‐1 and PlGF (from Roche, Germany) in the retained serum of the selected participants were detected by electrochemiluminescence.

### 2.3 Statistical analysis

All data were analyzed with SPSS21.0. Measurement data complying with the normal distribution were presented by means plus or minus standard deviation ($\overset{¯}{X}$ ± *s*). The differences between the two groups were analyzed by *t* test or Mann‐Whitney *U* test, and the chi‐square test was used to compare the rates between groups. The optimal cut‐off value, specificity and sensitivity of sFlt‐1, PlGF, and sFlt‐1/PlGF ratios for predicting preeclampsia were determined by the receiver operating characteristic curve. Values of *P* < 0.05 were considered statistically significant.

## 3 RESULTS

### 3.1 Clinical characteristics

There were 48 cases in preeclampsia group, with an average age of 32.5 ± 4.8 years and an average gestational age of 29.1 ± 3.1 weeks. The control group consisted of 134 patients, with an average age of 31.9 ± 4.3 years and an average gestational age of 28.5 ± 5.3 weeks. There was no difference in age, gestational age, BMI, number of pregnancy, or multipara percentage between the preeclampsia patient group and the control group (shown in Table 1).

**Table 1:** Clinical characteristics of the two groups

	Control group (n = 134）	Preeclampsia group (n = 48）	*t*/*χ* ^2^/*Z*	*P*
Age (y)	31.9 ± 4.3	32.5 ± 4.8	−0.802^a^	0.424
Gestational age (wk)	28.5 ± 5.3	29.1 ± 3.1	−0.744^b^	0.457
SBP (mm Hg)	118.6 ± 16.1	157.1 ± 24.6	−7.828^b^	<0.001
DBP (mm Hg)	77.3 ± 12.5	103.7 ± 16.6	−7.766^b^	<0.001
24 h urine protein (g/L)	0.07 ± 0.6	4.3 ± 3.9	−11.668^b^	<0.001
BMI (Kg/m^2^)	22.4 ± 3.6	22.8 ± 3.5	−0.552^a^	0.582
Number of pregnancy (times)	2.35 ± 1.14	2.70 ± 1.54	−1.625^b^	0.104
Multipara percentage (%)	46.1	46.2	0.003^c^	0.959

Age (y), Gestational age (wk), SBP (mm Hg), DBP (mm Hg), 24 h urine protein (g/L), BMI(Kg/m^2^), and Number of pregnancy (times) were presented by means ± SD.

a The *t* statistic to *t* test

b The *Z* statistic to Mann‐Whitney *U* test

c The *χ* ^2^ statistic to chi‐square test.

### 3.2 Comparison of observation indicators

Because the measurement data of the observed indicators are non‐normal distribution, nonparametric Mann‐Whitney *U* test was used for analysis. As shown in Table 2, comparing with the control group, the factors of sFlt‐1, P1GF, and sFlt‐1/PlGF were significantly different with PE group (shown in Table 2).

**Table 2:** Comparison of observation indicators of the two groups

	Control group (n = 134）	Preeclampsia group (n = 48）	*Z*	*P*
sFlt‐1(ng/L)	2153.8 ± 1458.4	12928 ± 11726.2	−7.021	<0.001
PlGF(ng/L)	422.2 ± 282.3	123.2 ± 221.7	−8.129	<0.001
sFlt‐1/PlGF	7.8 ± 9.5	474.2 ± 688.7	−8.583	<0.001

sFlt‐1(ng/L), PlGF(ng/L), and sFlt‐1/PlGF were presented by means ± SD.

**Figure 1:**
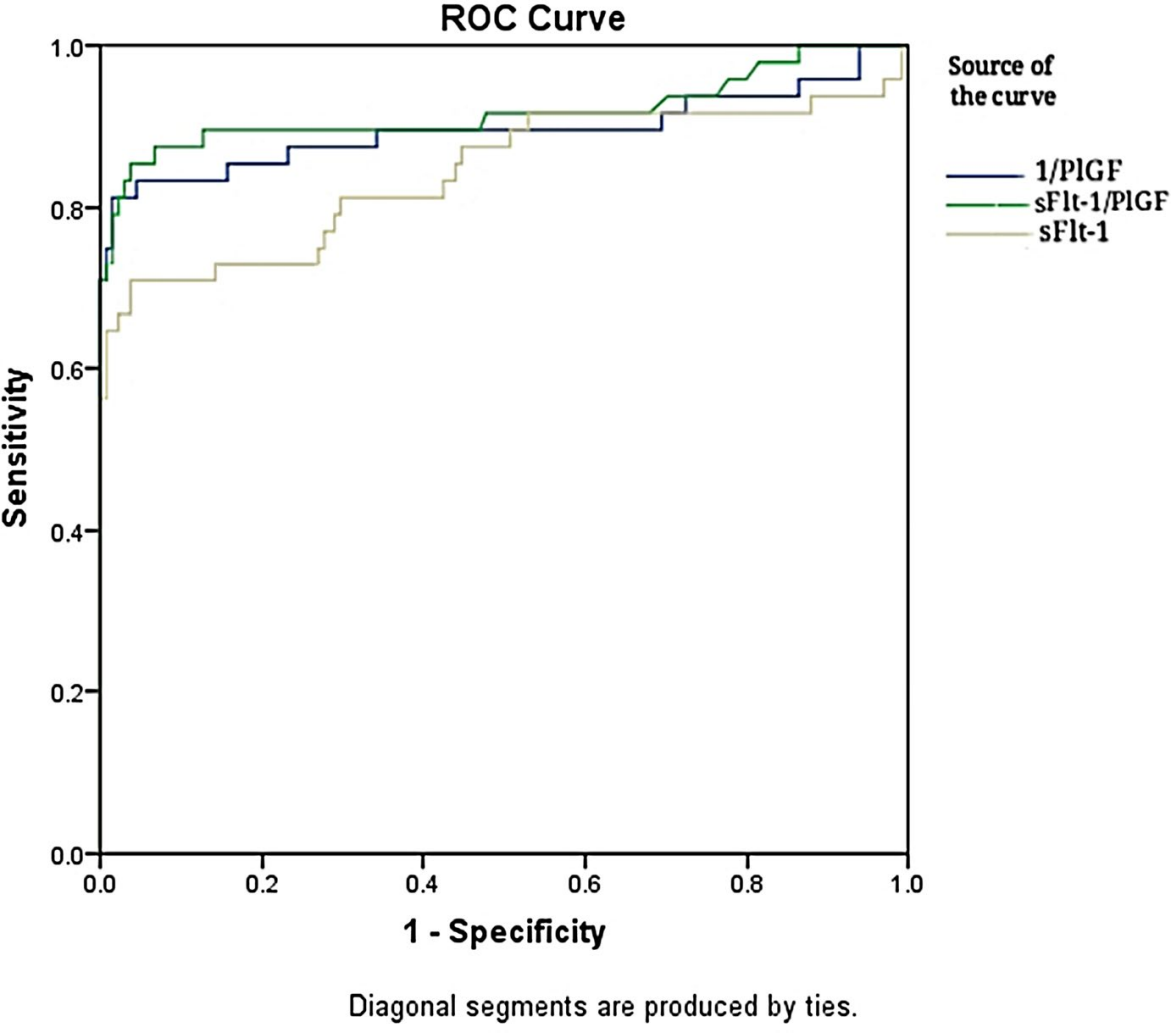
Analysis of receiver operating characteristic curve with sFlt‐1, PlGF, and sFlt‐1/PlGF ratio

Analysis of receiver operating characteristic curve (Figure 1).

Using sFlt‐1 to predict the preeclampsia alone and when the area under ROC curve (AUC) was 0.842 (0.805‐0.879), the sensitivity was 70.83%, the specificity was 96.27%, the positive predictive value was 87.2%, the negative predictive value was 90.2%, the positive likelihood ratio was 18.99, the negative likelihood ratio is 0.3, and the Youden index was 0.671 when cut‐off value was 4626 ng/L.

Inverse processing the PIGF data to predict the preeclampsia, when the area under the ROC curve (AUC) was 0.896 (0.863‐0.929), the sensitivity was 81.25%, the specificity was 98.51%, the positive predictive value was 95.1%, and the negative predictive value was 93.6%, the positive likelihood ratio was 54.5, the negative likelihood ratio was 0.19, and the Youden index was 0.797 when the cut‐off value was 90.8 ng/L.

Using sFlt‐1/PlGF ratio to predict the preeclampsia, when the area under ROC curve (AUC) was 0.918 (0.889‐0.946), the diagnostic sensitivity was 85.42%, the specificity was 96.27%, the positive predictive value was 89.1%, the negative predictive value was 94.9%, the positive likelihood ratio was 22.9, the negative likelihood ratio was 0.15, and the Youden index was 0.817 when cut‐off value was 26.6. As shown in Table 3, the SFlt‐1, PlGF, and sFlt‐1/PlGF ratios were significant in predicting preeclampsia. However, the predictive efficacy of sFlt‐1/PlGF ratio was significantly better than that of PlGF (*Z* value 2.070，*P* = 0.0385); but no difference with sFlt‐1 (*Z* value 1.713，*P* = 0.0867). (shown in Table 3).

**Table 3:** Predictive performance indicators for preeclampsia

	AUC	SEM	*P*	95% Confidence interval	
				Lower	Upper
sFlt‐1	0.842	0.019	<0.001	0.805	0.879
1/PlGF	0.896	0.017	<0.001	0.863	0.929
sFlt‐1/PlGF	0.918	0.015	<0.001	0.889	0.946

AUC, area under roc curve; SEM, standard error.

## 4 DISCUSSION

Preeclampsia (PE) is a specific multisystem disease that occurs after 20 weeks in gestation and is one of the main causes of maternal and neonatal death. The clinical manifestations of PE are hypertension, proteinuria, and/or edema. At present, its pathogenesis is still unclear.

For early‐onset PE, taking conservative treatment can reduce neonatal death caused by immature fetus; for late‐onset PE, pregnancy should be terminated. Therefore, early diagnosis of PE is extremely important for improving the prognosis of pregnant women and fetuses.

This study focused on the relationship between serum sFlt‐1 and PlGF levels with preeclampsia. Hertig et al found that the increasing serum sFlt‐1 at an average of 6.5 weeks earlier than the clinical symptoms by continuous detection.^6^ The sensitivity and specificity of predicting preeclampsia were 80% and 100%, respectively, when the threshold was 0.975 g/L. Lee et al found that the serum levels of sFlt‐1 was 934.5 ± 235.5 ng/L in preeclampsia patients and was 298.0 ± 168.2 ng/L in normal pregnancy control group.^7^ We assessed the serum levels of sFlt‐1 in pregnant women with medium‐term and late period of preeclampsia. The results showed that the serum levels of sFlt‐1 in preeclampsia group were significantly higher than that in control group (*P* < 0.05), and it indicates that the soluble fms‐like tyrosine kinase‐1 was a risk factor for preeclampsia, which is consistent with the results reported in the literature above.

Mijal et al found that the serum level of PlGF in preeclampsia pregnant women was significantly decreased by comparing with normal pregnant women.^8^ Cetin I et al found that PlGF level could be used to predict the risk associated with adverse pregnancy such as very low birthweight infants and emergency childbirth.^9^ However, our results showed that the level of serum PlGF in preeclampsia group was significantly lower than that in normal pregnancy control group (*P* < 0.05) and indicated that placental growth factor was a protective factor for preeclampsia. Many researches advocated that low level of PlGF in pregnant women could auxiliary diagnosis preeclampsia by specific binding sFlt‐1. Stepan et al and Verlohren et al found that predicted preeclampsia more accurately by combining sFlt‐1 and PlGF.^10^, ^11^ And the predictive specificity of early‐onset preeclampsia is 8.3%, and sensitivity is 95%. Maynard et al showed that the sFlt‐1/PlGF ratio could predict the occurrence of preeclampsia; sensitivity was 94% and specificity was 77%.^12^ Our study combined sFlt‐1 and PIGF and found that the ratio of sFlt‐1/PLGF in preeclampsia group was significantly difference from that in normal group (*P* < 0.05), which increased several dozen times. The data showed that the sFlt‐1/PLGF ratio was more sensitive than the single index. It was recommended that patients should seek medical help as soon as possible when sFlt‐1 increased, and/or PlGF decreased, especially the ratio of sFlt‐1/PlGF was increased gradually after repeated examinations. Another key objective of this study is using the receiver operating characteristics curve (ROC) to determine the best cut‐off value of sFlt‐1/PlGF ratio for predicting preeclampsia in a regional population. ROC curve was a method to evaluate the accuracy of diagnostic tests by combining sensitivity and specificity. Analyzing by the shape of the curve and the area under the curve, the result is not affected by the prevalence rate. In the last decade, with the emergence of more accurate and precise quantitative detection methods, many scholars have begun to focus on the sFlt‐1/PlGF ratio for the prediction and diagnosis of preeclampsia; Rana et al and Kleinrouweler et al believed that the serum sFlt‐1, PlGF, and sFlt‐1/PlGF ratios were the great value in the diagnosis of preeclampsia.^13^, ^14^ When the truncation value was 19.47, the sensitivity and specificity of sFlt‐1/PlGF were 80.6% and 82.4%, but the large‐scale clinical research are still lacking. A multicenter and prospective study for preeclampsia demonstrated that the sFlt‐1/PlGF ratio would reach a negative predictive value of 99.3%, sensitivity (80%), and specificity (78.3%) at a cut‐off point of 38 using a specific instrument initiated by the European Association of Obstetrics and Gynecology in 2016.^15^ Since race, heredity, living environment, and habits are also related factors in the pathogenesis of preeclampsia, each country and region should determine appropriate diagnostic boundaries according to its own population characteristics and should not copy the standards of other laboratories. In order to observe the predictive value of the sFlt‐1, PlGF, and sFlt‐1/PlGF, the time point of peripheral specimen collection was not confined but carried out together with the obstetrical examination of pregnant women. All peripheral blood sampling was completed before the occurrence of preeclampsia in pregnant women. The results of our study showed that when the optimal truncation value of ROC curve was 26.6, the area under the curve was 0.918, the sensitivity was 85.42%, the specificity was 96.27%, the positive predictive value was 89.1%, the negative predictive value was 94.9%, the positive likelihood ratio was 22.9, and the probability of positive results in preeclampsia patients was 22.9 times higher than that in normal patients. The negative likelihood ratio was 0.15, and the probability of detecting negative results in preeclampsia patients was only 0.15 times higher than that in normal patients. The sFlt‐1/PlGF ratio can be used as an indicator to predict the occurrence of preeclampsia because the rise of sFlt‐1/PlGF happens earlier than the diagnosis of preeclampsia. The preeclampsia can be diagnosed in the middle and late pregnancy, therefore the time lag between taking the samples and having the diagnosis of preeclampsia can be as short as a few days or as long as 2‐3 months. The application of this ratio can effectively avoid adverse effects on fetus and perinatal outcomes of pregnant women in the early diagnosis of preeclampsia. Our data proved that serum levels of sFlt‐1 and PlGF were independent risk factors for preeclampsia through a series of tests and evaluations, especially the determination of sFlt‐1/PlGF ratio was valuable to detect the occurrence of preeclampsia in advance, targeted detection of the sFlt‐1/PlGF ratio can play an important role in the prediction of pregnant women with preeclampsia. Clinic should take interventions treatment and preventive measures in advance when the ratio is a rising trend at continuously monitoring. The ratio and cut‐off value were used as indicators for routine screening. It can improve the accuracy of diagnosis and prediction of preeclampsia by combining them with clinical manifestations, pathological Doppler ultrasonography, and other laboratory tests.

## CONFLICTS OF INTEREST

The authors declare that they have no competing interests.

## AUTHORS’ CONTRIBUTIONS

Fan Yu was responsible for the statistical analysis and prepared the manuscript. Yongmei Jiang was responsible for the study design and coordination, guided the statistical analysis, and revised the manuscript. Shihong Zhang was responsible for the study design and coordination and reviewed the manuscript critically. Qianjin Bai collected the data and reviewed the manuscript. All authors read and approved the final manuscript. The excel data used to support the findings of this study are available from the corresponding author upon request.
